# Thoracic Manual Therapy With or Without Exercise Improves Pain and Disability in Subacromial Pain Syndrome: A Systematic Review of Randomized Trials

**DOI:** 10.3390/healthcare13192479

**Published:** 2025-09-29

**Authors:** Román Robles-Pérez, Rodrigo Vallejo-Martínez, Andoni Carrasco-Uribarren, Sandra Jiménez-del-Barrio, Héctor Hernández-Lázaro, Luis Ceballos-Laita

**Affiliations:** 1 Grupo de Investigación Clínica en Ciencias de la Salud, Facultad de Ciencias de la Salud, Universidad de Valladolid, 42004 Soria, Spain; roman.robles@uva.es (R.R.-P.); hector.hernandez.lazaro@uva.es (H.H.-L.); luis.ceballos@uva.es (L.C.-L.); 2 Facultad de Medicina y Ciencias de la Salud, Universitat International de Catalunya, 08195 Barcelona, Spain; acarrasco@uic.es

**Keywords:** subacromial pain syndrome, thoracic manual therapy, therapeutic exercise, randomized controlled trials, systematic review

## Abstract

Objectives: The aim of this systematic review was to evaluate the effectiveness of thoracic manual therapy with or without exercise for improving clinical outcomes (pain, disability, range of motion (ROM), quality of life (QoL) and satisfaction) in patients with subacromial pain syndrome (SPS). Methods: A systematic review was conducted following PRISMA guidelines. Randomized controlled trials (RCTs) involving thoracic manual therapy with or without thoracic exercise for patients with SPS were included. Databases searched included PubMed, PEDro, Cochrane Library, and Web of Science up to April 2025. The methodological quality was evaluated with the PEDro scale. Results: Seven RCTs involving 393 patients were included. Interventions ranged from thoracic manipulation alone to combinations with exercises. Better outcomes were reported for every clinical outcome evaluated: pain, disability, ROM, QoL and satisfaction. However, methodological heterogeneity and variability in follow-up durations limited result generalizability. Conclusions: Thoracic manual therapy applied in isolation or with exercise was reported to have positive effects in reducing pain and disability in patients with SPS, especially in the short term. These findings support the inclusion of thoracic interventions as complementary strategies in shoulder rehabilitation programs. Future high-quality trials with long-term follow-up are needed to confirm and standardize these approaches.

## 1. Introduction

Subacromial pain syndrome (SPS) is one of the leading causes of shoulder pain and dysfunction, accounting for up to 70% of consultations for shoulder pathology in primary care [1,2,3,4]. It is typically associated with pain during overhead movements, reduced range of motion (ROM), and functional limitation, and is believed to result from mechanical compression of the rotator cuff tendons and the subacromial bursa beneath the coracoacromial arch [5,6,7].

Conventional physiotherapy management includes rotator cuff strengthening, scapular motor control exercises, and manual therapy targeting the glenohumeral and scapulothoracic joints [8,9,10,11]. While these interventions are commonly used and have shown benefits in pain and function, persistent or recurrent symptoms have been reported in nearly 50% of cases, suggesting that standard approaches may not address all clinically relevant factors [7,12].

Emerging evidence indicates that proximal impairments, particularly thoracic spine dysfunction, may contribute to the persistence of symptoms in SPS. Thoracic hypomobility, excessive kyphosis, and limited thoracic extension have been associated with altered scapular kinematics, including increased anterior tilt and decreased upward rotation, which may reduce the subacromial space and increase mechanical compression [6,7,13]. In fact, a minimum of 10–15 thoracic extension is required to achieve full shoulder elevation [4,14]. Individuals with thoracic postural deviations exhibit a higher prevalence of rotator cuff injuries [15,16].

This perspective aligns with the concept of regional interdependence, which proposes that dysfunction in one region may influence the function of adjacent areas [7,12]. As a result, thoracic spine-focused interventions, including manual therapy and extension-based exercises, have gained attention as adjuncts to shoulder rehabilitation.

Several randomized controlled trials (RCTs) have reported that the addition of thoracic manual therapy to exercise-based programs results in superior improvements in pain, disability, ROM, and patient satisfaction compared to exercise alone [11,13,17]. Furthermore, systematic reviews and clinical studies support the short- and long-term benefits of thoracic-directed interventions, showing clinically relevant improvements in pain, functional limitations, and biomechanical dysfunctions such as reduced mobility or altered scapular kinematics [3,12,17].

Despite promising results, thoracic-focused interventions remain underused in clinical practice, in part due to heterogeneity in treatment protocols, variability in methodological quality, and lack of consensus on long-term efficacy [11,12,18]. Therefore, the aim of this systematic review is to determine the effectiveness of thoracic spine-focused interventions—such as high-velocity low-amplitude (HVLA) thrust manipulation, non-thrust mobilization, Mulligan sustained natural apophyseal glides (SNAGs), and muscle energy techniques (MET)—and/or active thoracic exercises mainly based on thoracic extension and posture-centered drills [19,20] in improving clinical outcomes, including pain, disability, range of motion (ROM), and strength, in individuals with shoulder impingement syndrome [21,22,23]. These findings aim to support future clinical recommendations and inform guideline development.

## 2. Materials and Methods

### 2.1. Study Design

This systematic review of RCTs was prepared and conducted according to the Preferred Reporting Items for Systematic Reviews (PRISMA) guidelines [24] and Cochrane’s recommendation for systematic reviews [17]. The study protocol was pre-registered in PROSPERO under the unique identification number (CRD420251008587).

### 2.2. Search Strategy

Electronic searches were performed across PubMed (MEDLINE), PEDro, the Cochrane Library (CENTRAL), and Web of Science (Core Collection) for records dated 1 January 2015–30 April 2025, restricted to English and Spanish reports in human adults.

For search strategy, Medical Subject Headlines (MESH) terms were combined with different keywords including shoulder impingement syndrome, rotator cuff, exercise movement techniques, exercise therapy, thoracic spine mobilization, pain measurement and RCT. Furthermore, boolean operators AND, OR were used; the specific search strategy was defined as shown in Appendix A. Search strings, database-specific syntax, record counts, and search dates are provided in Appendix A.1 and Appendix A.2.

### 2.3. Eligibility Criteria

Eligibility was defined a priori using the PICOS framework [17,18]. Only RCTs were considered.

- Population. Adults with a clinical diagnosis of SPS.
- Interventions. Thoracic-directed care: manual therapy with or without exercise applied to the thoracic spine, delivered either as a stand-alone intervention or as an adjunct to standard non-pharmacological conservative care.
- Comparators. Sham procedures, no treatment/usual care, or the same standard conservative care without the thoracic component.
- Outcomes. Primary: pain and shoulder-related disability. Secondary: shoulder range of motion (ROM), health-related quality of life (QoL), and patient satisfaction (Global Rating of Change, GROC).
- Study design. RCTs.
- Filters. Language and timeframe: English or Spanish; January 2015 to April 2025.

Exclusion criteria. Non-RCTs; post-surgical populations; adhesive capsulitis; cervical-only interventions without a thoracic component; non-shoulder conditions.

### 2.4. Study Selection

**Figure 1:**
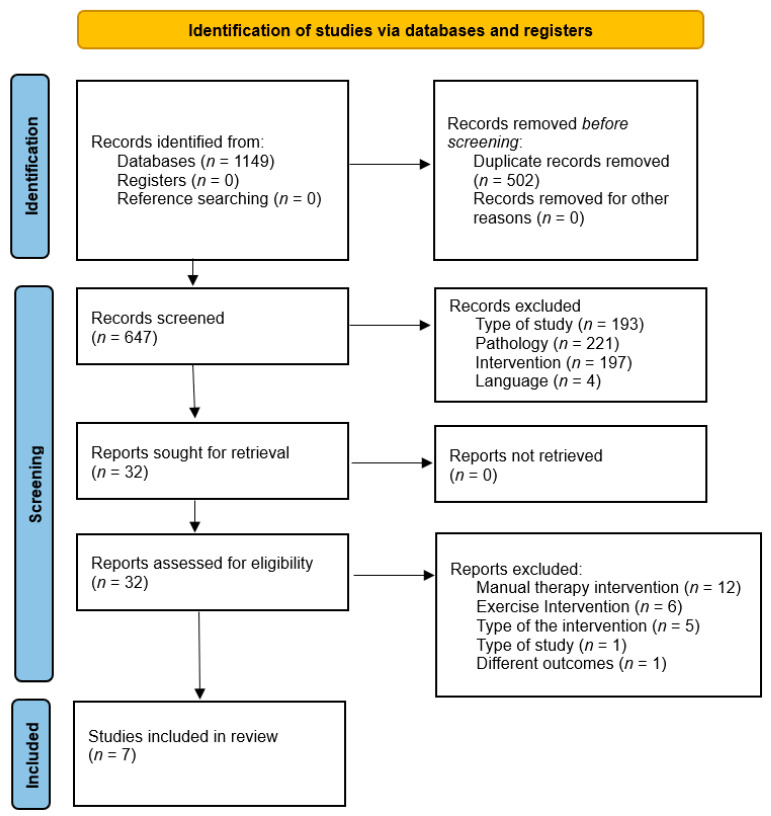
PRISMA 2020 flow diagram for new systematic reviews, including searches of databases and registers only.

All records retrieved from the databases were exported to Mendeley, where duplicates were identified and removed. The PRISMA flow diagram (Figure 1) also documents additional records located through manual searching of reference lists and relevant reviews. Two reviewers (RR and RV) independently screened titles and abstracts against the PICOS criteria. Articles considered potentially eligible were obtained in full text for assessment. Any disagreements were resolved by a third reviewer (LC). Reasons for full-text exclusion are shown in Figure 1.

### 2.5. Data Extraction

Two reviewers (RR and RV) independently extracted data using a piloted form developed from Cochrane Collaboration guidance. Items recorded included study and population characteristics (diagnosis, sample size, mean age), and intervention details (technique description, session duration, weekly frequency, and total number of sessions). Technique-level descriptors of thoracic manual therapy (e.g., HVLA target levels, SNAGs (extension or rotation), mobilization grades) were also captured and are summarized in Appendix B. When a trial contained more than one intervention arm, arms were coded as A and B within the same RCT.

### 2.6. Methodological Quality Assessment

Methodological quality was appraised independently by two reviewers (RR and RV) using the PEDro scale [25], an 11-item checklist derived through a Delphi process. Scores were assigned separately by each reviewer, and any discrepancies were settled by consensus or, if needed, by a third reviewer.

The PEDro scale judges trial rigor across domains such as randomization, allocation concealment, blinding, and statistical reporting. Total scores were interpreted as follows: 0–3 = poor; 4–5 = fair; 6–8 = good; ≥9 = excellent. In accordance with PEDro guidance, Item 1 (eligibility criteria/external validity) was not counted in the total; the remaining 10 items address internal validity and interpretability.

## 3. Results

Our searches retrieved 1149 hits; after eliminating the 502 duplicates, 647 titles and abstracts were reviewed, and 32 RCTs were selected for full-text review. Seven articles were included in the review [11,13,26,27,28,29,30]. Twelve studies were excluded because although they used manual therapy, they did not target the thoracic spine [31,32,33,34,35,36,37,38,39,40,41,42]; six studies were excluded because they only used exercise therapy without focusing on the thoracic spine [43,44,45,46,47,48]; one was excluded because of the type of study; another was excluded because of the type of outcomes they measured [49]. Five studies were excluded because of the type of intervention they used; the study by Nazary et al. [50] was excluded because the intervention was not exclusively focused on the thoracic region for the treatment of SPS; the studies conducted by Sharma et al. [8,9,51] were excluded because they compared two interventions without including a control group; finally, the study of Dunning et al. [10] was excluded because the intervention combined thoracic, cervical, and rib manipulation with dry needling, making it impossible to isolate and extrapolate the specific effects of thoracic spine mobilization. A description of the selection process is shown in the PRISMA flowchart diagram (Figure 1).

### 3.1. Characteristics of the Included Studies

A total of seven RCTs (*n* = 393) were included. Studies are presented and tabulated by year of publication. Most studies included individuals with symptoms lasting at least one month and a clinical diagnosis of SPS confirmed by at least two positive orthopedic tests, such as Neer, Hawkins-Kennedy, Empty Can, Painful Arc, or External Rotation Resistance Test [11,13,26,27,28,29,30]. Sociodemographic and clinical characteristics of the included participants are summarized in Table 1.

Studies that applied thoracic manual therapy in isolation compared thoracic interventions with sham manipulation [26,27,29] or soft tissue techniques compared with placebo laser. Studies that combined thoracic manual therapy and exercise compared thoracic interventions with shoulder exercises versus shoulder exercise in isolation [11,13,28,30]. The presence of exercise in the control group allowed for extrapolation of the results of the thoracic spine interventions. Detailed information about the tecnhniques can be consulted in Appendix B. A detailed description of the interventions is provided in Table 2.

In line with our prespecified PICOS, the primary outcomes across trials were pain and shoulder-related disability, and the secondary outcomes were shoulder ROM, QoL, and patient-reported satisfaction. Pain was assessed with the Visual Analogue Scale (VAS); disability with the Shoulder Pain and Disability Index (SPADI) and the Disabilities of the Arm, Shoulder and Hand (DASH), including the QuickDASH short form where reported [11,28,29]. ROM was measured using goniometry; QoL with the Western Ontario Rotator Cuff Index (WORC) or study-specific instruments; and satisfaction with Global Rating of Change scales (GROC) [13,29,30].

### 3.2. Methodological Quality

The methodological quality of the studies included was evaluated using the PEDro scale. One study achieved an “excellent” score ≥ 9 points [27], five studies scored between 6 and 8 points, corresponding to a “good” quality level [11,13,26,28,29], and one was classified as “fair” [30]. Overall, the body of evidence reflects moderate to high methodological quality, with a consistent trend favoring structured interventions that include thoracic manual therapy. The PEDro scale scores for all studies are shown in Table 3.

## 4. Discussion

This review of RCTs examined thoracic manual therapy in isolation or combined with active thoracic exercises for adults with SPS. According to the presented evidence, a clear pattern emerges. Thoracic-directed care can help in the short term, especially for pain and disability, but the reliability and size of the effect depend on how the intervention is implemented. Programs that used manual therapy in isolation rarely showed consistent superiority over comparators [26,27,29]. In contrast, when manual therapy was integrated with thoracic exercise, improvements in pain and disability were more frequent and clinically meaningful [11,13,28,30,52].

These differences are plausible. Manual techniques may provide a rapid hypoalgesic effect and restore segmental mobility, but such effects can be transient. Adding extension- and posture-oriented thoracic drills likely consolidates mobility gains, increases exposure to pain-free movement, and improves scapulothoracic coordination. These mechanisms are consistent with models of regional interdependence linking thoracic hypomobility and altered alignment to suboptimal shoulder mechanics [8,9,29]. The combined approach therefore appears to act through complementary biomechanical and neurophysiological pathways, which helps explain why it outperforms stand-alone manipulation in several trials.

A decrease in pain intensity and disability was observed in all the studies, although the combination of thoracic manual therapy with therapeutic exercise achieved better results than using thoracic manual therapy alone. On the one hand, Hunter et al. B [27] reported improvements in pain as compared with a placebo laser, whereas arm A compared with MET techniques showed no between-group advantages, aligned with the results showed by Grimes et al. [29] that showed small between-group differences and did not reach the minimal clinically important difference. Haik et al. [26] also reported within-group changes without clear between-group benefits. On the other hand, interventions that combined manual therapy with therapeutic exercise had stronger and more consistent effects; Park et al. [28] and Abu El Kassem et al. [13] found that thoracic mobilization plus extension exercises reduced pain intensity and disability.

Beyond the primary outcomes, the ROM improvement was modest and most evident when an active thoracic component accompanied manual therapy. Isolated techniques seldom changed ROM in a meaningful way [13,28]. Findings for QoL and patient satisfaction were sparse and mixed, reflecting heterogeneity in instruments (e.g., WORC or study-specific QoL scales; different GROC anchors) and short follow-up windows [11,27]. In practical terms, clinicians can expect short-term reductions in pain and disability with thoracic-focused programs, may observe small ROM gains, and should interpret QoL/satisfaction changes cautiously until stronger evidence accrues.

From a clinical standpoint, thoracic assessment and treatment should be viewed as an adjunct rather than a stand-alone solution. For patients with evident thoracic stiffness or excessive kyphosis, multimodal plans that combine thoracic manual therapy with structured thoracic exercises are more likely to yield meaningful short-term benefits than manipulation alone. Given the transient effects reported after single visits [26,27,29], multi-session dosing with home practice of thoracic extension drills and clear expectations about likely benefits is advisable.

Several factors temper confidence in these conclusions. Trials varied widely in technique, dose, and comparator content; many enrolled small samples, used incomplete blinding, or did not report between-group mean differences with confidence intervals, limiting precision. Most outcomes were assessed at short-term time points, and more than one study originated from the same research groups, which may restrict external validity. At the review level, restricting inclusion to English/Spanish reports within 2015–2025 may have missed relevant evidence.

Future work should prioritize pre-registered, multicentred RCTs with standardized thoracic protocols and dosing and a harmonized core outcome set. Careful exploration of dose–response relationships and of patient subgroups (e.g., marked thoracic hypomobility/kyphosis) will help identify who benefits most and how durable the effects are.

## 5. Conclusions

Thoracic-directed interventions such as manual therapy, applied in isolation or combined with active thoracic exercises, are associated with short-term, clinically meaningful improvements in pain and disability in adults with SPS. Small gains in shoulder ROM may be observed, whereas effects on QoL and patient satisfaction are inconsistent across trials. On the current evidence, thoracic care should be implemented as an adjunct to rehabilitation rather than as a stand-alone treatment. Integrating mobility and posture-oriented thoracic strategies is biologically plausible to optimize scapulothoracic mechanics and support symptom reduction, but confirmation requires stronger evidence.

## Figures and Tables

**Table 1:** Characteristics of the included studies and main results.

Participants				Intervention				
Author (Year)	Country	Mean Age (SD)	Population	Thoracic Extension Exercises	Control Group	Session Duration	Frequency (Sessions/Week)	Total Number of Sessions
Thoracic manual therapy in isolation								
Haik et al. (2017) [26]	Brazil	EG: 32.5 (12.0) CG: 31.3 (11.0)	Adults with SPS *n* = 61	Thoracic spine manipulation *n* = 30	Sham TSM *n* = 31	NR	2	2
Grimes et al. (2019) A [29]	United States	EG: 37.6 (15.3) CG: 36.5 (15.5)	Adults with SPS *n* = 40	Supine thrust manipulation *n* = 20	Sham manipulation *n* = 20	2 sessions	1	1
Grimes et al. (2019) B [29]	United States	EG: 35.6 (14.7) CG: 36.5 (15.5)	Adults with SPS *n* = 40	Seated thrust manipulation *n* = 20	Sham manipulation *n* = 20	2 sessions	1	1
Hunter et al. (2022) A [27]	Australia	EG:62.0 (9.6) CG: 61.4 (11.3)	Adults 40+ with SPS *n* = 50	MET *n* = 25	Placebo laser *n* = 25	15 min	1	4
Hunter et al. (2022) B [27]	Australia	EG: 56.9 (9.2) CG: 61.4 (11.3)	Adults 40+ with SPS *n* = 50	MET + Soft Tissue Mobilization *n* = 25	Placebo laser *n* = 25	15 min	1	4
Thoracic manual therapy combined with exercise								
Haider et al. (2018) [30]	Pakistan	EG: 49.3 (9.9) CG: 49.8 (9.7)	Adults with SPS *n* = 40	Thoracic manipulative therapy + exercise *n* = 20	Exercise therapy *n* = 20	Not described	3	6
Park et al. (2020) A [28]	Korea	EG: 49.2 (9.48) CG: 50.2 (8.99)	Adults with SPS and thoracic kyphosis *n* = 20	Thoracic mobilization + extension exercises *n* = 10	Thoracic Mobilization *n* = 10	15 min	3	12
Park et al. (2020) B [28]	Korea	EG: 50.9 (9.10) CG: 50.2 (8.99)	Adults with SPS and thoracic kyphosis *n* = 20	Thoracic mobilization + extension exercises *n* = 10	Extension exercises *n* = 10	15 min	3	12
Abu El Kassem et al. (2024) [13]	Egypt	EG: 32.15 (4.93) CG: 31.6 (4.59)	Recreational adults with SPS *n* = 74	SNAGs + shoulder exercises *n* = 37	Traditional shoulder exercises *n* = 37	60 min	3	12
Michener et al. (2024) [11]	United States	EG: 52.3 (13.3) CG: 54.0 (11.1)	Adults with SPS *n* = 93	Thoracic & scapular manual therapy + resistance exercise *n* = 52	Resistance exercise *n* = 41	45–60 min	2	10

SD: Standard deviation EG; Experimental group; CG: Control group; SPS: Subacromial pain syndrome; TSM: Thoracic Spinal Manipulation; MET: Muscle Energy Therapy; NR: Not reported.

**Table 2:** Characteristics of the interventions.

Author (Year)	Intervention	Control Group	Outcome (Tool)	Intragroup Results—MD (SD)	Between Groups MD (95% CI)
Thoracic manual therapy in isolation					
Haik et al. (2017) [26]	Thoracic manipulation	Sham manipulation	Pain (VAS) Disability (DASH) Quality of life (WORC)	NR −9.75 (no SD) −13.9% (no SD)	−1.1 (−1.7 to −0.5) * +0.1(−2.5 to 2.8) * −5.0 (−9.7 to −0.3) *
Grimes et al. (2019) A [29]	Thoracic supine manipulation	Sham manipulation	Pain and function (PSS)	+15.2 ± 4.8	+4.0 (−2.0 to 5.8) *
Grimes et al. (2019) B [29]	Thoracic seated manipulation	Sham manipulation	Pain and function (PSS)	+13.6 ± 4.4	+2.0 (−0.5 to 5.3) *
Hunter et al. (2022) A [27]	Muscle Energy Tecnhique (MET) + Soft tissue massage (STM)	MET	Pain and Disability (SPADI) Disability (DASH) Pain (VAS) Satisfaction (GROC)	NR NR NR NR	−1.1 (9.8 to 7.5) * −0.26 (6.0 to 5.5) * −7.7 (16.8 to 1.5) * 0.3 (0.4 to 1.0) *
Hunter et al. (2022) B [27]	MET + STM	Placebo laser	Pain and Disability (SPADI) Disability (DASH) Pain (VAS) Satisfaction GROC)	NR NR NR NR NR	−13.5 (22.3 to 4.8) * −8.2 (14.0 to 2.3) * −7.8 (17.1 to 1.5) * −2.0 (2.3 to 0,3) * +1.2 (0.5 to 1.9) *
Thoracic manual therapy combined with exercise					
Haider et al. (2018) [30]	Non-thrust + 3 thrust manipulations	Shoulder exercises	Pain (NPRS) Pain and Disability (SPADI)	−0.70 ± 0.92 −12.3 ± 4.7	−4.65 (−15.2 to −5.8) * + 12.2*
Park et al. (2020) A [28]	Thoracic mobilization	Thoracic mobilization + Shoulder exercises	Pain and Disability (SPADI) ROM: (Goniometer): External rotation (ER) Internal rotation (IR)	−8.2 ± 3.1 NR NR	−5.13 * +2.7 * +2.7 (2.09 to 3.24) *
Park et al. (2020) B [28]	Shoulder exercises	Thoracic mobilization + Shoulder exercises	Pain and Disability (SPADI) ROM: ER IR	−6.7 ± 2.9 NR NR	−5.77 * 2.0 * 2.3 *
Abu El Kassem et al. (2024) [13]	SNAGs + Shoulder exercises	Shoulder exercises	Pain and Disability (SPADI) Pain (VAS) ROM (Goniometer): ER IR	−24.2 ± 8.7 −4.4 ± 1.3 NR NR	1.61 (9.88 to 16.73) * 1.3 (3.65 to 5.56) * +5.7 (25.88 to 10.51) * +7.1 (26.57 to 7.42) *
Michener et al. (2024) [11]	Thoracic + scapular manual therapy + Shoulder exercises	Shoulder exercises	Disability (DASH) Satisfaction (GROC)	−6.7 ± 3.5 +2.3 ± 1.5	−6.7 (11.4 to 2.1) * NR

* *p* value > 0.05 in favor of the intervention group; MD: mean difference; SD: Standard deviation; CI: confidence interval; NR: not reported; VAS: Visual Analogue Scale; DASH: Disabilities of the Arm, Shoulder and Hand; WORC: Western Ontario Rotator Cuff Index; PSS: Patient Satisfaction Scale; MET: muscle energy technique; STM: Soft tissue massage; SPADI: Shoulder Pain and Disability Index; ROM: range of motion; ER: external rotation; IR: internal rotation; SNAGs: sustained natural apophyseal glides; GROC: Global Rating of Change; NPRS: Numeric Pain Rating Scale.

**Table 3:** PEDro scale.

Author (Year)	Item 1	Item 2	Item 3	Item 4	Item 5	Item 6	Item 7	Item 8	Item 9	Item 10	Item 11	Total PEDro Score (0–10)
Hunter et al. (2022) [27]	Y	Y	Y	Y	Y	N	Y	Y	Y	Y	Y	9
Haik et al. (2017) [26]	Y	Y	N	Y	Y	N	Y	Y	Y	Y	Y	8
Abu El Kassem et al. (2024) [13]	Y	Y	Y	Y	N	N	Y	Y	Y	Y	Y	8
Michener et al. (2024) [11]	Y	Y	Y	Y	N	N	Y	Y	Y	Y	Y	8
Park et al. (2020) [28]	Y	Y	Y	Y	N	N	Y	Y	N	Y	Y	7
Grimes et al. (2019) [29]	Y	Y	Y	N	N	N	Y	Y	N	Y	Y	6
Haider et al. (2023) [30]	N	Y	N	Y	N	N	N	Y	N	Y	Y	5

Item 1: Eligibility criteria described; Item 2: Random allocation; Item 3: Concealed allocation; Item 4: Baseline comparability; Item 5: Blind subjects; Item 6: Blind therapists; Item 7: Blind assessors; Item 8: Adequate follow-up; Item 9: Intention-to-treat analysis; Item 10: Between-group comparisons; Item 11: Point estimates and variability. Note: Eligibility criteria item does not contribute to total score.

## Data Availability

No new data were created or analyzed in this study.

## Abbreviations

The following abbreviations are used in this manuscript:

AHD	acromiohumeral distance
DASH	Disabilities of the Arm, Shoulder and Hand
EMG	electromyography
GROC	Global Rating of Change
HHD	handheld dynamometry
HVLA	high-velocity low-amplitude
MCID	minimal clinically important difference
MeSH	Medical Subject Headings
NPRS	Numeric Pain Rating Scale
PEDro	Physiotherapy Evidence Database scale
PRISMA	Preferred Reporting Items for Systematic Reviews and Meta-Analyses
QoL	quality of life
RCT	randomized controlled trial
ROM	range of motion
SNAGs	sustained natural apophyseal glides
SPADI	Shoulder Pain and Disability Index
SPS	Subacromial Pain Syndrome
TSM	thoracic spine manipulation
TSTM	thoracic spine thrust manipulation
VAS	Visual Analogue Scale
WoS	Web of Science
WORC	Western Ontario Rotator Cuff Index

## Appendix A. Detailed Search Strategy According to PRISMA and Model Search Terms

### Appendix A.1. Search Terms

**Population**	**Intervention**	**Results**	**Type of Study**
Shoulder Impingement Syndrome (MeSH)	Exercise Movement Techniques (MeSH)	Pain Measurement (MeSH)	Randomized Controlled Trial (MeSH)
Rotator Cuff (MeSH)	Exercise Therapy (MeSH)	Pain (MeSH)	Randomized Clinical Trial
Rotator Cuff Tendinopathy	Thoracic Spine Mobilization	Disability Evaluation (MeSH)	
Subacromial Shoulder Pain	Exercise Therapy	Pain Intensity	
	Thoracic Manual Therapy	Symptom	
	Therapeutic Exercise	Disability	

### Appendix A.2. Search Strategy

#### Appendix A.2.1. PUBMED

((((Shoulder Impingement Syndrome[MeSH Terms]) OR (Rotator Cuff[MeSH Terms]) OR (“Shoulder Impingement Syndrome”) OR (“Rotator Cuff”) OR (“Rotator Cuff Tendinopathy”) OR (“Subacromial Shoulder Pain”)) AND (AND (Exercise Movement Techniques[MeSH Terms]) OR (Exercise Therapy[MeSH Terms]) OR (“Exercise Movement Techniques”) OR (“Exercise Therapy”) OR (“Thoracic Spine Mobilization”) OR (“Therapeutic Exercise”) OR (“Thoracic Manual Therapy”) OR (“Exercise Therapy”))) AND ((Pain Measurement[MeSH Terms]) OR (Pain[MeSH Terms]) OR (Disability Evaluation[MeSH Terms]) OR (“Pain Measurement”) OR (“Pain”) OR (“Disability Evaluation”) OR (“Pain Intensity”) OR (“Symptom”) OR (“Disability”))) AND ((Randomized Controlled Trial[MeSH Terms]) OR (“Randomized Controlled Trial”) OR (“Randomized Clinical Trial”))

Results: 186

Filter: 10 years

Date: 30 April 2025

#### Appendix A.2.2. PEDro

**First strategy:** exercise therapy AND shoulder impingement syndrome

Results 48

**Second strategy:** thoracic spine mobilization AND shoulder impingement syndrome

Results 2

**Third strategy:** exercise therapy AND shoulder pain syndrome

Results 69

**Fourth strategy:** thoracic spine mobilization AND shoulder pain syndrome

Results: 121

Date: 30 April 2025

#### Appendix A.2.3. Cochrane Library

(Shoulder Impingement Syndrome) OR (Rotator Cuff) OR (Rotator Cuff Tendinopathy) OR (Subacromial Shoulder Pain) AND (Exercise Movement Techniques) OR (Exercise Therapy) OR (Thoracic Spine Mobilization) OR (Therapeutic Exercise) OR (Thoracic Manual Therapy) OR (Exercise Therapy) AND (Pain Measurement) OR (Pain) OR (Disability Evaluation) OR (Pain Intensity) OR (Symptom) OR (Disability) AND (Randomized Controlled Trial) OR (Randomized Clinical Trial)

Results: 341

Filter: 10 years

Date: 30 April 2025

#### Appendix A.2.4. Web of Science

(Shoulder Impingement Syndrome) OR (Rotator Cuff) OR (Rotator Cuff Tendinopathy) OR (Subacromial Shoulder Pain) AND (Exercise Movement Techniques) OR (Exercise Therapy) OR (Thoracic Spine Mobilization) OR (Therapeutic Exercise) OR (Thoracic Manual Therapy) OR (Exercise Therapy) AND (Pain Measurement) OR (Pain) OR (Disability Evaluation) OR (Pain Intensity) OR (Symptom) OR (Disability) AND (Randomized Controlled Trial) OR (Randomized Clinical Trial)

Results: 500

Filter: 10 years

Date: 30 April 2025

## Appendix B. Detailed Techniques

**Author (Year)**	**Intervention**	**Technique Details**	**Control Group**
Thoracic manual therapy in isolation			
Haik et al. (2017) [26]	Thoracic manipulation	Middle thoracic spine, patient seated with arms crossed over chest. The therapist was located behind the patient and performed a thrust technique with arms and chest around the thoracic region of the subject. Sham: Same position without performing thrust. The technique was applied twice in a period of 3 to 4 days apart. Sham technique was previously reported as a believable active treatment.	Sham manipulation
Grimes et al. (2019) A [29]	Thoracic manipulation	One of the three interventions was delivered between the levels of C7 and T4, and was performed two times on each participant based on methods used in previous studies. For the supine TSTM, examiner used his body to push down through the participant’s upper arms to provide a high-velocity, low-amplitude thrust in the anterior-to-posterior direction. Sham: Seated manipulation moving the participant through the same motion but delivering no manipulative thrust. This sham technique has been previously validated as a plausible treatment.	Sham manipulation
Grimes et al. (2019) B [29]	Thoracic manipulation	One of the three interventions was delivered between the levels of C7 and T4, and was performed two times on each participant based on methods used in previous studies. For the seated TSTM, examiner applied a high-velocity, low-amplitude distraction thrust in a cephalad direction Sham: Same seated sham manipulation	Sham manipulation
Hunter et al. (2022) A [27]	Muscle Energy Tecnhique (MET)	MET consisted of the application of lateral force to the spinous process of the thoracic vertebra until initial resistance with the vertebra below was noted.	MET
Hunter et al. (2022) B [27]	MET + Soft tissue massage (STM)	Same MET technique. STM was applied to the rotator cuff (subscapularis, infraspinatus, and teres minor) and the triceps muscle of the affected shoulder. The STM techniques included static compression and deep longitudinal stripping to the aforementioned muscles, with active engagement.	Placebo laser
Thoracic manual therapy combined with exercise			
Haider et al. (2018) [30]	Non-thrust + 3 thrust manipulations	In thoracic group, thoracic manipulative therapy included one non-thrust mobilization and three different thrust manipulation techniques directed at thoracic spine and exercise therapy including hot or cold pack, mobility exercises (flexion and extension exercises with arms in front of the wall, shoulder flexion 90°, and exercises with shoulder circles) and strengthening exercises (resistance exercise with elbow flexion 90° and an elastic band, shoulder flexion with elbow extension holding bar (1–4 kg), body lift from a seated position with elbows extended, and resistance exercises for external rotation. In group 2, patients received conservative exercise therapy including hot or cold pack, mobility exercises and strengthening exercises.	Shoulder exercises
Park et al. (2020) A [28]	Thoracic mobilization	Thoracic spine joint mobilization consisted of oscillatory techniques performed in the prone position, with 30 repetitions per set, four sets in total, and a 1-min rest between sets. Central posterior–anterior mobilization was applied using a grade III large-amplitude rhythmic oscillation, targeting the joint sign segment (the most painful or stiffest level) identified through Maitland’s passive accessory intervertebral motion test. When a joint sign was not present, mobilization was applied at T6–T7. The mobilization session lasted 15 min. The exercise program aimed to enhance thoracic spine extension, trunk extensor strength, and trunk flexor flexibility. It included foam roll stretches as a warm-up, marching on a roller (2 sets of 10 repetitions), thoracic extension against a wall using bodyweight (2 sets of 10 repetitions), and a standing neck/chest stretch as a cool-down. Each exercise session lasted 15 min.	Shoulder exercises
Park et al. (2020) B [28]	Thoracic Mobilization + Shoulder exercises	The combination group received an intervention consisting of joint mobilization and an exercise program. Joint mobilization involved central posterior–anterior oscillations performed in the prone position for 30 repetitions, with a 1-min rest between 2 sets. The exercise program included foam roll stretches (warm-up), marching on a roller (1 set of 10 repetitions), thoracic extension against a wall using bodyweight (1 set of 10 repetitions), and a standing neck/chest stretch (cool-down). The total duration of the combined therapy was 15 min, equally divided between joint mobilization (7 min 30 s) and exercise (7 min 30 s).	Shoulder exercises
Abu El Kassem et al. (2024) [13]	SNAGs + Shoulder Exercises	SNAGs technique on the thoracic spine. The patient sat at the far end of the table, hands behind his neck, protracting the scapulae, and providing the therapist’s hand accessibility to the mid-thoracic spine. The therapist did stand on their most effective side for a centrally administered SNAG. Firstly, a restricted segment of the thoracic spine was detected. The therapist performed passive extension of the thoracic spine and at the same time made palpation of the thoracic spinous process to detect the restricted segment of the thoracic spine. The therapist’s mobilizing hand (ulnar border) was used to apply a cephalad glide in line with the facet joint plane of the involved spinal level, while the other arm held the thoracic wall above the level to be mobilized. The patient was then asked to perform thoracic extension while gliding was applied to the restricted thoracic segment to the end of the range. Traction was administered before glide, which was accomplished via the therapist’s knee extension. The technique was repeated for 6 to 10 times for 3–5 sets with rest in between. Shoulder exercises included pendulum exercises, shoulder range of motion exercises, stretching exercises, strengthening exercises and scapular stabilization exercises.	Shoulder Exercises
Michener et al. (2024) [11]	Thoracic + scapular manual therapy + Shoulder exercises	Manual therapy combined both thrust manipulation and non-thrust mobilization. The manual therapy techniques were aimed at three areas. Clinicians were instructed to apply manual techniques for a total duration of 10–15 min, ensuring the use of at least one technique in each of the following regions: thoracic spine, posterior shoulder, and glenohumeral joint. Practitioners could select low-grade techniques for those with moderate to high irritability, whereas high-grade techniques could be used for those with low irritability. Thoracic techniques were thoracic PA glides in prone, thoracic PA glides seated, thoracic thrust in prone (max 2 reps), thoracic thrust in supine (max 2 reps) and distraction thrust (max 2 reps). Shoulder exercises combined progressive resistance exercise with stretching. The program consisted of resistance exercises using body weight or exercise bands to target the shoulder muscles with particular emphasis on the scapular stabilizers and rotator cuff, complemented with flexibility exercises and postural training through chin tucks and scapular retraction. Strengthening exercises were performed using latex-free Thera-Bands, with 2 to 3 sets of 10 repetitions.	Shoulder exercises
